# Understanding overweight and obesity subgroups: a cluster analysis of data from the UK Yorkshire Health Study

**DOI:** 10.1186/s12889-025-24152-7

**Published:** 2025-09-30

**Authors:** Rachel O’Hara, John Stephenson, Elizabeth Goyder, Sara Eastburn, Hannah Jordan

**Affiliations:** 1 Sheffield Centre for Health and Related Research (SCHARR), University of Sheffield, Sheffield, UK; 2 School of Human and Health Sciences, University of Huddersfield, Huddersfield, UK

**Keywords:** Weight status, Overweight, Obesity, Body mass index, Classification, Cluster analysis, Yorkshire Health Study

## Abstract

**Background:**

Individuals with overweight/obesity are a heterogeneous population and a better understanding of factors differentiating subgroups can help deliver more targeted weight management interventions that benefit everyone equally. Previous research employed cluster analysis to understand heterogeneity within a population with obesity in one region of England, using the Yorkshire Health Study (YHS) dataset. The aim of this study is to build on that research and contribute a more detailed understanding of subgroups to support more tailored weight management strategies.

**Methods:**

The study entailed using cluster analysis methods to identify a number of discrete subgroups characterised by demographic, health and lifestyle commonalities, using a larger Yorkshire Health Study (YHS) dataset (*n* = 47,080) and broader range of weight categories (healthy weight, overweight and obesity). Clustering involved using the k-prototypes method for mixed data types and the optimum number of clusters was determined by identifying the point of inflexion (elbow) on the scree plot.

**Results:**

Six-clusters were identified as the optimum overall solution, which comprised six distinct subgroups differentiated by a range of variables related to weight status: younger, healthy, active, heavy drinking males; older with poor physical health, but good quality of life; older with poor health, quality of life and well-being; older, ex-smokers with poor health but high well-being; younger, healthy and active females; and younger with poor mental health and well-being.

**Conclusions:**

The findings contribute additional insight on differences between specific population groups in relation to key determinants of weight. This understanding should ensure that within an overall systems based approach to tackling this major public health issue, there is adequate attention to delivering more tailored weight management strategies for different groups.

## Introduction

The continuing trend for increasing rates of obesity represents an ongoing public health challenge, both globally and within the UK. The worldwide prevalence of obesity has more than doubled since 1990 [1] and within the UK, it is estimated that over half of the adult population could be obese by 2050 [2]. The World Health Organization (WHO) defines both obesity and overweight as conditions of excessive fat deposits and health risk factors [1]. Body Mass Index (BMI) is widely used as an indicator of body fat, employing a calculation of weight [kg] divided by height squared [m²] to distinguish between healthy weight (18.5 kg/m^2^ ≤ BMI < 25 kg/m^2^) overweight (25 kg/m^2^ ≤ BMI < 30 kg/m^2^) and obesity (BMI ≥ 30 kg/m^2^) [3]. Despite criticism regarding the precision of BMI as an estimate of body fat [4, 5], it remains useful as a population-level measure [6]. 

The individual level impact of obesity is significant in terms of poorer physical and mental health, quality of life and life expectancy [7–11]. Relatedly, it is estimated that UK healthcare costs associated with overweight and obesity will double to £10 billion per year by 2050 [2]. The economic impact at an individual and societal level is also projected to increase due to the effects of poor health on employment and sickness absence [12, 13]. There is a wide variation in the prevalence of overweight and obesity in different population groups and the associated health impacts, which is factor in widening health inequalities [14]. The intersectional nature of these inequalities means that a much more nuanced understanding of predictors of body weight is required to identify the relative importance of different factors for different populations and individuals in order to inform the development of effective intervention strategies.

To date, strategies intended to reduce adult obesity at a population level have been unsuccessful, both globally and within the UK. For example, a ‘call to action’ by the UK government was intended to reduce levels of excess weight across adults by 2020 [15] but rates of both overweight and obesity have continued to increase, with 64% of adults in 2023 classified as overweight or living with obesity [16]. The UK Government’s Foresight programme acknowledged that it would take several decades to reverse the complex and multifaceted factors contributing to obesity trends [2]. The Foresight report advocates a whole systems approach to understanding and addressing obesity by mapping inter-related factors associated with weight status, which do not necessarily act in isolation, but may amplify or mitigate health outcomes by interacting with one another [2, 17]. Morris et al.(2018) recommend that the comprehensive scope of the UK Foresight obesity map provides a potential framework for combining data from different sources to inform potential interventions, that addresses the lack of a single data source and cost of primary data collection [18]. 

Previous research shows that individuals with overweight and obesity are a heterogeneous population and highlights the need for a better understanding of the factors differentiating subgroups, to support a more targeted approach to weight management interventions [19–21]. Green et al.(2015) employed cluster analysis to understand heterogeneity within a population with obesity in one region of England, using the Yorkshire Health Study (YHS) dataset [19]. Their analysis identified six distinct subgroups– ‘heavy drinking males’, ‘young healthy females’, ‘the affluent and healthy elderly’, ‘the physically sick but happy elderly’, ‘the unhappy and anxious middle aged’, and ‘those in poorest health’. However, their analysis was limited to participants classified as having obesity and did not reference relevant Foresight obesity system variables (e.g. level of employment, functional fitness, physical activity, alcohol consumption, smoking cessation, stress, reliance on medicines) [17]. Clark et al.(2022) similarly conducted a cluster analysis using UK Biobank data in England, the variables in the analysis were mapped to a number of the Foresight obesity system variables and included different weight categories, not just obesity. They identified eight subgroups distinguished by their exposure to known drivers of obesity [20]. 

This study builds on the research by Green et al. [19] and aims to contribute a more detailed understanding of population subgroups by using cluster analysis methods to identify a number of discrete subgroups characterised by demographic, health and lifestyle commonalities. The insight obtained will complement existing evidence to support the delivery of more tailored weight management strategies for different groups to potentially reduce weight related health inequalities. The research entails using a larger, more representative Yorkshire Health Study (YHS) dataset [22] and broader range of weight categories (healthy weight, overweight and obesity) than Green et al.(2015) in the analysis of variables associated with weight status, which are also mapped to relevant Foresight variables [17].

## Methods

### Data collection

Data was extracted from the YHS, a longitudinal observational study that collected information on personal, lifestyle and health factors in the Yorkshire and Humberside (Y&H) region of England between 2010 and 2015. The complete dataset used for this study was derived from two separate phases of data collection that gathered self-reported data on sociodemographics, lifestyle and health-related variables, including long-term conditions and health service utilisation, from a total sample of 70,836 individuals. The Phase 1 sample included 27,813 participants recruited via general practitioner (GP) surgeries between 2010 and 2012. The Phase 2 sample provided an additional 43,023 participants more representative of the regional population, recruited via an online regional media campaign and National Health Service (NHS) Trusts from 2013 to 2015. The sample has been summarized descriptively elsewhere, by phase of data collection and as a full cohort [22, 23]. The sample for this study includes participants recruited in Phases 1 and 2. Some variables, for example income and dietary habits, were only included in Phase 2 and were therefore excluded from analysis. Only variables available in both phases were included. Individuals included in the analysis were adults aged 18 and over and those excluded were those who were pregnant or underweight (BMI < 18.5 kg/m^2^). Pregnant individuals (*n* = 1,768; 2.5%) were excluded to minimise potential bias due to the influence of pregnancy on included variables, in particular BMI but also many of the other variables, including health service attendance, health status and lifestyle behaviour. Underweight individuals (*n* = 535; 0.8%) were excluded as the contributory factors are potentially different and the aim of the study is to support the targeting of weight management strategies to reduce overweight/obesity and increase healthy weight. A total sample of 47,080 from the overall cohort has been included in this study to provide the most complete data set for analysis.

### Data analysis

The current analysis extends the approach of Green et al.(2015) which used the Phase 1 sample only to identify subgroups within participants classified as having obesity [19]. It includes the complete data set from both phases of data collection and the wider population of people with healthy weight, overweight and obesity. Variables used to identify clusters were consistent with those used by Green et al.(2015) where data was available from both phases of the data collection. Additional details of variables are provided in Table 1.

**Table 1:** Variables included in the cluster analysis

Variable	Details	Foresight themes– variables [17]
Age	Years	Not included in the Foresight systems map
Gender	Male; Female; Other/Not given	Not included in the Foresight systems map
Index of Multiple Deprivation (IMD) score	Index of multiple deprivation for postcode location	Food production - Purchasing power
Employment status	Employed; Not-employed	Food production - Level of employment
Body mass index (BMI) score	Weight (kg)/Height (m^2^)	Nodal variable - Energy balance (measurable basis for overweight and obesity).
Health-related quality of life - EQ-5D score	EuroQol 5 Dimension (EQ-5D) Quality of Life measure derived from 5 component scale scores (mobility, self-care, activities, pain and anxiety)	Individual activity - Functional fitness
Well-being - Life satisfaction score	Single-item measure on 11-point scale of how satisfied participants were with their lives (0 = very dissatisfied, 10 = very satisfied)	Individual psychology - Self esteem, stress
Physical health Long Term Conditions (LTCs)	Number of physical health conditions reported (pain, diabetes, breathing problems, hypertension, osteoarthritis)	Individual activity - Functional fitness
Mental health Long Term Conditions (LTCs)	Number of mental health conditions reported (anxiety, depression, fatigue, insomnia)	Individual psychology - Stress, self esteem
Serious illness/Long Term Conditions (LTCs)	Number of severe conditions reported (heart disease, stroke, cancer)	Individual activity - Functional fitness
Days off work, household tasks, and leisure activities due to health (last 3 months)	None; 1 to 3; 4 to 9; 10 to 29; 30+ (unique quantile cuts)	
Primary care attendance (last 3 months)	None; 1 to 2; 3; 4 + (unique quantile cuts)	Physiology/Individual psychology - Reliance on medicines, pharmacological remedies, surgical remedies
Secondary care attendance (last 3 months)	None; 1 to 2; 3; 4 + (unique quantile cuts)	
Mental health service attendance (last 3 months)	None; 1 to 3; 4 to 6; 7 + (unique quantile cuts)	Individual psychology - Stress, self esteem
Physical health service attendance (last 3 months)	None; 1 to 2; 3 to 4; 5 + (unique quantile cuts)	Physiology/Individual psychology - Reliance on medicines, pharmacological remedies
Social care attendance (last 3 months)	None; 1 to 3; 4 to 11; 12 + (unique quantile cuts)	Individual activity - Functional fitness
Alternative care attendance (last 3 months)	None; once; 2 to 3; 4 + (unique quantile cuts)	Individual psychology/Physiology - Reliance on medicines, Self esteem
Smoking status	never smoked; used to smoke occasionally; used to smoke daily; smoke occasionally but not every day; smoke daily	Social psychology - Smoking cessation
Alcohol consumption (units per week)	Below 14 units per week; 14–27 units per week; 28–41 units per week; More than 42 units per week	Food consumption - Alcohol consumption
Walking (hours per week)	None; <1 hour; 1-2 hours; 3+ hours	Individual activity - Physical activity
Physical exercise (hours per week) e.g. running, swimming, football, gym, cycling.	None; Some activity (<1 hour); 1 activity (1-3 hours); >1activity (>2 hours); >1 activity (>4 hours)	Individual activity - Level of recreational activity

Sociodemographic variables included age, gender, socioeconomic deprivation and employment status. Ethnicity data was not included as this was only available for Phase 1. An Index of Multiple Deprivation (IMD) score was determined on the basis of individual postcode location to provide a multidimensional measure of area deprivation [24]. Individual BMI scores were also included for each participant. Health-related quality of life was measured using the EuroQoL EQ-5D and general well-being was assessed by asking individuals to rate how satisfied they were with their life [25]. Health status data included whether an individual reported experiencing any of 12 long-term conditions (pain, diabetes, breathing problems, hypertension, osteoarthritis, anxiety, depression, fatigue, insomnia, heart disease, stroke, cancer) that were combined to create three long-term condition (LTC) variables (physical health, mental health and severe illness) due to low numbers for many of these individual conditions (see Table 1 for details of conditions in each variable). The impact of health on number of days off work, household tasks and leisure activities was assessed as a single variable for these activities combined, and for behaviour in relation to attendance at various health care services. Lifestyle behaviours included smoking status, units of alcohol consumed, level of walking activity and level of engagement in other physical activity. Green et al.(2015) used dichotomous/binary data for lifestyle and health behaviour variables whereas the current analysis has used interval data (i.e. recorded on a scale in which differences between values are meaningful and equal) in order to provide a higher level of detail. Health service usage/attendance data were transformed using quantiles to divide observations into more comparable ordinal variables. Table 1 also identifies variables included in the analysis that potentially map onto six of the seven Foresight obesity system themes and associated variables [17]. 

#### Cluster analysis

Cluster analysis was conducted to explore subgroups of individuals with similar characteristics across the range of variables identified in Table 1 [26]. The clustering method employed was different to that of Green et al.(2015) to accommodate the analysis of a larger dataset and mixed-type data comprising numerical and categorical variables [27]. Clustering involved using the k-prototypes method for mixed data types [28, 29]. This method is faster and computationally less demanding compared to full hierarchical clustering [30]. K-prototype cluster results were generated for 2 to 10 cluster solutions using Gower distances, which computes the distance between observations weighted by variable type, and takes the mean across all variables [26]. Five random start points were used for each solution, selecting the result with lowest total distances in each case.

A scree plot of the sum of all observations’ distances to their corresponding cluster prototype was produced. There is no definitive method for identifying the best number of clusters [26, 31] although the elbow method is commonly used with K-prototypes clustering to identify the point at which there is unlikely to be any value in additional clusters, by identifying step changes or static points in the graph of distance measure against increasing k-values [32]. Therefore, the point of inflexion (elbow) on the scree plot curve was used as an indicator of the optimum number of cluster divisions.

All analyses were undertaken using R version 4.4.0 (2024-04-24) [33] and the scree plot was produced in ggplot2 version 3.5.1 [34].

## Results

Table 2 presents the sociodemographic characteristics of the sample.

**Table 2:** Descriptive summary of sample sociodemographic characteristics

Sociodemographic variable	*N* (%)
Gender (*n* = 46,998) Male Female	39.4% 60.6%
Age group (*n* = 47,080) 18–24 25–34 35–44 45–54 55–64 65–74 75 +	8.9% 12.0% 13.8% 18.2% 19.7% 17.9% 9.6%
Deprivation quintile (*n* = 47,080) 1 (Least deprived) 2 3 4 5 (Most deprived)	22.3% 20.6% 17.5% 18.0% 21.7%
Employment status (*n* = 47,080) Employed Not employed	52.8% 47.2%
BMI category (*n* = 47,080) Healthy weight (18 kg/m2 ≤ BMI < 25 kg/m2) Overweight (25 kg/m2 ≤ BMI < 30 kg/m2) Obesity (30 kg/m2 ≤ BMI < 40 kg/m2) Severe obesity (BMI ≥ 40 kg/m2)	43.1% 35.0% 19.3% 2.5%

**Fig. 1:**
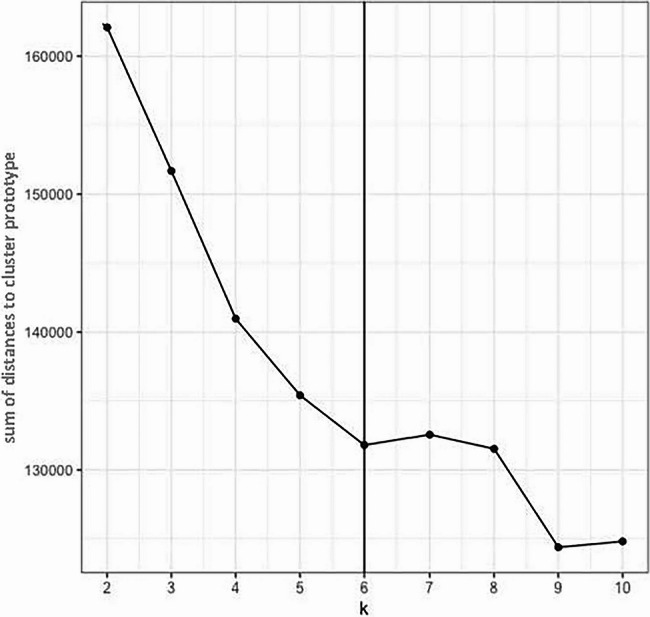
Scree (elbow) plot– optimal number of clusters A line graph (scree plot) of the sum of all observations’ distances to their corresponding cluster prototype showing distance measure on the y axis and number of clusters on the x axis, with a distinct point of inflexion (elbow) at six clusters

The scree plot of the sum of all observations’ distances to their corresponding cluster prototype is presented in Fig. 1. The elbow appears most prominently at six clusters; which was selected as the optimum overall solution for the analysis. Six clusters was also regarded as providing a parsimonious solution in terms of maximising information and differences, but minimising the complexity of having too many groups.

Table 3 provides cluster characteristics according to the cluster variables (means/proportions). Table 4 provides supplementary detail for some of these variables in the form of sub-categories for BMI classification, EQ-5D subscales and individual long term conditions in order to aid interpretation.

**Table 3:** Cluster characteristics by cluster variables

Variables*	Cluster 1	Cluster 2	Cluster 3	Cluster 4	Cluster 5	Cluster 6	All
Sample size	19.3% (9,085)	22.1% (10,427)	9.6% (4,531)	14.6% (6,858)	24.3% (11,430)	10.1% (4,749)	100% (47,080)
Age	44.27 (15.13)	60.30 (17.80)	58.89 (17.17)	65.74 (13.84)	40.78 (13.06)	43.19 (14.04)	51.40 (18.13)
Proportion male	100% (9,066)	15.5% (1,609)	24.8% (1,120)	88.4% (6,051)	0.0% (0)	14.6% (694)	39.4% (18,540)
Proportion female	0.0% (0)	84.5% (8,799)	75.2% (3,400)	11.6% (797)	100% (11,415)	85.4% (4,047)	60.6% (28,458)
Deprivation (IMD) score	20.98 (16.46)	21.46 (16.98)	28.86 (18.96)	23.72 (17.60)	20.40 (15.68)	22.67 (16.77)	22.28 (17.02)
Proportion in employment	83.0% (7,540)	7.4% (772)	15.4% (699)	10.9% (750)	93.8% (10,716)	92.1% (4,372)	52.8% (24,849)
Body mass index (BMI) score	26.35 (4.80)	26.60 (5.78)	29.55 (7.15)	27.49 (4.79)	25.63 (5.20)	27.05 (5.84)	26.78 (5.59)
Quality of life - EQ-5D score	0.90 (0.14)	0.82 (0.19)	0.46 (0.33)	0.75(0.24)	0.91 (0.12)	0.78 (0.19)	0.81 (0.23)
Well-being - life satisfaction score	7.64 (1.67)	7.82 (1.82)	5.82 (2.40)	7.68 (1.91)	7.63 (1.59)	6.90 (1.82)	7.43 (1.91)
Proportion with physical health LTC	24.2% (2,199)	46.3% (4,825)	88.6% (4,013)	69.0% (4,735)	18.2% (2,075)	40.7% (1,931)	42.0% (19,778)
Proportion with mental health LTC	11.9% (1,085)	24.4% (2,543)	80.8% (3,662)	29.9% (2,048)	15.4% (1,756)	37.4% (1,776)	27.3% (12,870)
Proportion with severe health LTC	3.3% (300)	10.2% (1,065)	25.7% (1,166)	24.9% (1,710)	1.3% (148)	5.7% (273)	9.9% (4,662)
Proportion having days off work, household tasks, and leisure activities due to health (last 3 months)	17.1% (1,549)	10.5% (1,100)	74.4% (3,373)	14.1% (970)	15.5% (1,773)	76.5% (3,634)	26.3% (12,399)
Proportion attending primary care (last 3 months)	33.9% (3,076)	66.1% (6,891)	87.8% (3,978)	75.0% (5,143)	37.3% (4,267)	86.7% (4,119)	58.4% (27,474)
Proportion attending secondary care (last 3 months)	14.1% (1,281)	18.0% (1,878)	76.6% (3,469)	58.9% (4,037)	12.0% (1,366)	64.5% (3,064)	32.1% (15,095)
Proportion attending physical health care (last 3 months)	5.7% (520)	6.7% (699)	20.4% (925)	8.6% (592)	6.1% (701)	14.3% (680)	8.7% (4,117)
Proportion attending mental health care (last 3 months)	1.9% (176)	2.5% (262)	12.1% (547)	2.3% (160)	2.7% (313)	7.8% (369)	3.9% (1,827)
Proportion attending social care (last 3 months)	0.3% (26)	0.7% (77)	4.9% (223)	1.1% (75)	0.3% (29)	0.5% (24)	1.0% (454)
Proportion attending alternative care (last 3 months)	2.6% (236)	3.5% (369)	5.6% (252)	3.6% (245)	3.4% (393)	6.5% (308)	3.8% (1,803)
Proportion who smoke	13.2% (1203)	11.0% (1142)	17.4% (789)	11.9% (815)	11.6% (1330)	19.0% (900)	13.1% (6180)
Proportion who previously smoked	21.5% (1,951)	23.0% (2398)	27.6% (1251)	72.5% (4971)	20.7% (2361)	54.0% (2566)	32.9% (15,498)
Proportion consuming <14 units of alcohol per week	21.1% (1,913)	38.7% (4,031)	66.9% (3,030)	26.6% (1,824)	21.3% (2,429)	18.8% (893)	30% (14,120)
Proportion consuming > 27 units of alcohol per week	33.7% (3,060)	10.0% (1,047)	7.0% (319)	21.2% (1,455)	21.0% (2,403)	26.2% (1,242)	20.2% (9,526)
Proportion walking <1 hour per week	22.7% (2,059)	23.4% (2,441)	61.2% (2,772)	24.2% (1,658)	17.8% (2,033)	11.6% (551)	24.5% (11,514)
Proportion doing <1 hour physical exercise per week	43.7% (3,971)	78.6% (8,193)	90.4% (4,094)	79.6% (5,459)	51.4% (5,877)	58.5% (2,780)	64.5% (30,374)

*Numerical = Mean (*SD*), Categorical = Percentage (*N*)

**Table 4:** Cluster characteristics for cluster variable sub-categories - Percentage (N)

Variables	Cluster 1	Cluster 2	Cluster 3	Cluster 4	Cluster 5	Cluster 6	All
**BMI classification**							
Healthy weight (18 kg/m^2^ ≤ BMI < 25 kg/m^2^)	43.0% (3,907)	43.9% (4,582)	28.2% (1,277)	31.2% (2,141)	55.3% (6,320)	43.6% (2,071)	43.1% (20,298)
Overweight (25 kg/m^2^ ≤ BMI < 30 kg/m^2^)	40.2% (3,655)	35.0% (3,654)	31.1% (1,407)	44.4% (3,044)	28.3% (3,233)	31.6% (1,499)	35.0% (16,492)
Obesity (30 kg/m^2^ ≤ BMI < 40 kg/m^2^)	15.6% (1,420)	18.8% (1,965)	33.0% (1,497)	22.8% (1,562)	14.6% (1,665)	21.0% (995)	19.3% (9,104)
Severe obesity (BMI ≥ 40 kg/m^2^)	1.1% (103)	2.2% (226)	7.7% (350)	1.6% (111)	1.9% (212)	3.9% (184)	2.5% (1,186)
**EQ-5D subscales**							
Proportion with mobility problems (walking)	8.9% (808)	26.8% (2,793)	79.1% (3,586)	43.6% (2,992)	5.8% (668)	23.8% (1,129)	25.4% (11,976)
Proportion with self-care problems (washing/dressing)	1.6% (145)	5.5% (577)	48.8% (2,031)	12.8% (879)	0.6% (65)	5.4% (258)	8.4% (3,955)
Proportion with problems doing usual activities	8.8% (802)	23.3% (2,428)	81.3% (3,685)	39.5% (2,706)	5.8% (667)	30.7% (1,456)	24.9% (11,744)
Proportion with pain/discomfort	30.4% (2,762)	52.9% (5,512)	90.4% (4,098)	67.8% (4,648)	27.7% (3,161)	61.8% (2,937)	49.1% (23,118)
Proportion anxious/depressed	21.7% (1,970)	28.9% (3,015)	70.7% (3,204)	28.8% (1,978)	26.2% (2,989)	48.7% (2,312)	32.9% (15,468)
**Individual long term conditions**							
Proportion with breathing problems	6.3% (570)	9.8% (1,022)	30.3% (1,371)	17.2% (1,183)	6.2% (703)	10.0% (476)	11.3% (5,325)
Proportion with diabetes	4.1% (370)	7.2% (747)	21.0% (953)	17.1% (1,175)	1.9% (215)	4.5% (215)	7.8% (3,675)
Proportion with hypertension	9.1% (827)	22.9% (2,388)	36.9% (1,674)	35.4% (2,427)	4.5% (517)	9.5% (452)	17.6% (8,285)
Proportion with anxiety	4.9% (446)	9.8% (1,019)	40.1% (1,819)	9.0% (619)	7.3% (830)	18.7% (887)	11.9% (5,620)
Proportion with depression	4.2% (384)	6.6% (688)	37.8% (1,711)	8.0% (552)	5.5% (623)	15.6% (740)	10.0% (4,698)
Proportion with tiredness/fatigue	6.7% (606)	13.7% (1,425)	64.1% (2,906)	21.4% (1,468)	7.4% (843)	22.2% (1,053)	17.6% (8,301)
Proportion with insomnia	2.2% (202)	6.7% (696)	29.8% (1,349)	7.2% (491)	3.0% (344)	8.0% (381)	7.4% (3463)
Proportion with osteoarthritis	2.1% (193)	11.7% (1,223)	31.3% (1,416)	14.7% (1,007)	2.2% (247)	6.2% (295)	9.3% (4381)
Proportion with pain	8.7% (786)	18.7% (1,951)	69.9% (3,165)	30.8% (2,109)	7.2% (821)	25.0% (1,189)	21.3% (10,021)
Proportion with heart disease	1.9% (169)	5.6% (585)	15.2% (690)	15.4% (1,054)	0.4% (47)	2.2% (104)	5.6% (2649)
Proportion with cancer	1.0% (94)	3.6% (373)	8.7% (392)	8.9% (607)	0.7% (80)	3.1% (147)	3.6% (1,693)
Proportion with stroke	0.6% (53)	1.8% (192)	6.3% (284)	4.0% (277)	0.2% (25)	0.8% (36)	1.8% (867)

The following is a description of each cluster with the sample size per cluster (proportion, number) identified in brackets:

- Cluster 1 (19%, 9,085): Younger, healthy, active, heavy drinking males

This cluster comprised exclusively male participants and is one of the younger groups, with a high proportion in employment. It has the second lowest BMI score, with most members of either healthy weight or overweight with relatively low levels of obesity. Cluster members are the most physically active of those of any group and have among the highest quality of life and life satisfaction scores. They generally have good physical and mental health with low incidences of LTCs, and lowest use of all health services. The proportion of smokers was moderate relative to other clusters and a large proportion consume considerably higher levels of alcohol than recommended by the UK Government (<14 units a week), with a high proportion consuming more than 27 units per week.

- Cluster 2 (22%, 10,427): Older with poor physical health, but good quality of life and well-being

This cluster is the oldest of the groups and comprises a high proportion of female participants (85%), with only a very small proportion in employment. It includes the second highest proportion of participants with healthy weight; with a moderate proportion overweight and relatively low proportion having obesity. Cluster members have high rates of physical LTCs and primary care use relative to other conditions and services, which is also reflected in a high proportion reporting pain/discomfort, but nonetheless they reported a reasonably high quality of life and highest well-being of all groups. A high proportion participate in walking but otherwise report less physical activity. This group has the lowest proportion of smokers and the second lowest level of alcohol consumption.

- Cluster 3 (10%, 4,531): Older with poor health, quality of life and well-being

This is one of the smaller clusters and comprises an older population with a high proportion of female participants (75%). A relatively low proportion of cluster members are in employment and the mean deprivation score is notably higher than in other clusters. This group has the highest BMI score, with 72% of participants classified as overweight/obesity. Cluster members have substantially lower quality of life and life satisfaction scores, as well as markedly higher levels of LTCs and health service use, compared to members of other identified clusters. A very high proportion report problems engaging in usual activities: this is one of the least physically active groups. This group has the second highest proportion of smokers, but the lowest levels of alcohol consumption.

- Cluster 4 (15%, 6,858): Older, ex-smokers with poor health but high well-being

This cluster comprised an older population with a high proportion of males (88%) and low numbers in employment. Most of this group are overweight with a quarter classified as having obesity. It is notable that this group has very high proportion of participants who are ex-smokers (72%), though alcohol consumption levels are high. The incidence of physical LTCs is high, with more moderate levels of mental health and severe LTCs. This group reports the highest levels of heart disease, and second highest levels of diabetes and hypertension, with high usage of primary and secondary care. Group members report an impact on daily activities and relatively low quality of life but high life satisfaction. A high proportion participate in walking, but they engage less in physical exercise.

- Cluster 5 (24%, 11,430): Younger, healthy and active females

This is the largest cluster, comprised exclusively of female participants. It is the youngest group of the cohort, with the highest level of employment and lowest deprivation score. This group has the lowest mean BMI score and most members are at a healthy weight. Members are the most physically active of any group that includes females and have among the highest quality of life and life satisfaction scores of any identified clusters. Members generally have good physical and mental health, with low incidences of LTCs and low usage of health services. The proportion of smokers in the group is relatively low but alcohol consumption levels are high.

- Cluster 6 (10%, 4,749): Younger, with poor mental health and well-being

This is one of the smaller clusters, with a high proportion of younger females (85%) and a high level of employment. Most members of this group are overweight, with a quarter having obesity. Moderate levels of physical and mental health LTCs are reported relative to other clusters but the incidence of mental health conditions (anxiety, depression, fatigue) is higher than in most other clusters and healthcare use is high. Health related days off work/activities are highest for this group, which has low quality of life and particularly low life satisfaction. It has the highest proportion of all groups consuming ≥ 14 units of alcohol per week and smoking, as well as the second highest proportion of ex-smokers (54%). Most group members engage in walking, but to a much lesser extent in other forms of physical activity.

## Discussion

The analysis carried out identified six distinct subgroups that are differentiated by a range of variables related to weight status. Three clusters (C) comprise a younger population with relatively higher levels of employment (C1, C5, C6) and alcohol consumption. Two of these (C1, C5) are self-similar in many respects except that they are comprised either exclusively of male (C1) or female (C5) participants. They have lower BMI scores, and higher levels of quality of life, well-being and physical activity. In contrast, C6, which comprises predominantly female participants, has relatively higher levels of overweight/obesity; and much poorer health, quality of life and well-being. The other three clusters comprise an older population with generally poorer physical health and a lower proportion in employment. C3 has a higher proportion of female participants and is a clear outlier in having the highest deprivation score of all groups, along with much poorer health, quality of life and well-being, but the lowest alcohol consumption. C2 and C4 are more self-similar, with poor physical health but high well-being; they comprise either a substantial majority of female (C2) or male (C4) participants. C4 has higher levels of overweight/obesity and poorer health, quality of life and well-being relative to C2, as well as higher levels of smoking and alcohol consumption, but a markedly higher proportion of previous smokers than any other cluster.

The findings are consistent with previous studies showing that individuals with overweight and obesity are a heterogeneous population and a better understanding of the factors differentiating subgroups is needed for a more targeted approach to weight management interventions [19–21]. Studies in the UK and internationally have employed classification analysis to identify population subgroups [19–21, 35–37]. Even though the findings are not easily comparable across these studies due to sample differences (e.g. weight categories, age, cluster variables and sample size), they do provide useful complementary and comparative evidence. The UK Foresight obesity system map identifies a range of inter-related factors associated with weight status and provides a potential framework for combining data from different sources [17]. This paper builds on previous research by Green et al.(2015) that identified six distinct subgroups of individuals with obesity in the UK YHS dataset, by using an augmented dataset, wider range of weight categories and linking to relevant Foresight variables.

The findings provide insight on heterogeneity in relation to weight status beyond the distinction between specific weight categories. For example, cluster 4 has a notably higher proportion of ex-smokers, which may indicate they are more amenable to lifestyle changes. There appears to be some consistency with the obesity (BMI > 30 kg/m^2^) subgroups identified by Green et al. [19]. For example, both studies identified a group with particularly poor health, well-being and quality of life, along with higher BMI and deprivation scores, suggesting that for this sub-population, weight status may not be the immediate priority for health-related intervention. [19] This appears to indicate that the clusters identified in the Green et al.(2015) study were not unique to people living with obesity and are actually more general across the population, therefore it is worth considering that obesity is a condition which exists substantively across multiple population clusters.

The identification of age and gender as key variables in differentiating subgroups is consistent with findings from a similar classification analysis of UK Biobank data [20]. Other literature exploring obesity prevention and management strategies has also highlighted gender related heterogeneity [38–41]. Consideration of age-related variation is generally limited to the distinction between children/adolescents and adults rather than different life stages within the adult population. Relatedly, clusters C1 and C5 are distinguished as being similar in age (both comprise a relatively younger cohort), but different in their gender composition C1 is exclusively males and C5 is exclusively females). A focus on lifestyle/dietary change may be warranted for both groups, to minimise the risk of weight gain associated with alcohol consumption, as well as other alcohol related health problems. Promoting the healthy lifestyle behaviours that many are currently engaging in (e.g. physical activity) to maintain healthy weight or reduce excess weight as they age and have less time could facilitate healthy aging. There are potential gender considerations regarding the form of interventions (e.g. health education/promotion) that would be most effective, which could be explored through engagement with individuals representative of these clusters.

Despite the apparent importance of age and gender, they are not included in the Foresight obesity system map. The authors suggest the framework can be segmented according to these and other individual level variables (ethnicity, socioeconomic status) [17], whereas Clark et al.(2022) advocate encompassing them within the system map. Either approach would at least support a more nuanced approach to intervention [20]. Relatedly, this study illustrates the scope for mapping existing data sets to the Foresight obesity system themes and variables to identify heterogeneous subgroups and the most appropriate weight management interventions.

There is increasing recognition of the complexity of factors influencing weight and that the implementation of weight management interventions have not always benefitted everyone equally, for example, men are less likely to engage with weight management services [14]. This has focused attention on the need for approaches that are more individualised, as well as co-produced strategies. Findings from the current and similar studies examining subgroup diversity can support the design of weight management interventions by identifying specific individual characteristics influencing weight status to create more appropriate services and better engagement [42].

### Limitations

The YHS data is not very recent (2010–2015), in common with the Biobank data (2006 and 2010) used by Clarke et al., which also had a more limited age range (40 to 70 years old). However, the research using these data sets addresses the need identified by Morris et al. [18] for a combination of data from different sources to support a whole systems approach to understanding and addressing obesity. The YHS population is drawn from one geographical region and cannot be considered representative of the UK or other populations. Data on ethnicity was not recorded for the phase 2 participants; however, the phase 1 sample comprised a predominantly white population, and the overall proportion of ethnic minority participants is therefore likely to be similar or lower than the regional prevalence of 14.5% [43]. The proportion of females is high and even higher than the Green et al.(2015) sample, which seems to reflect an increased proportion of female participants in the second phase of data collection [22]. Further work is recommended to understand the extent to which the features of the subgroups identified in this study are shared across a more diverse range of populations and geographical locations.

The YHS data are based on self-reported information and are therefore subject to a range of different biases. The YHS variables are more focused on individual level factors that may influence weight status, which was also noted as a limitation of the UK Biobank data such that Foresight themes relating to individual behaviours were easier to map to the data than environmental, societal and food production [20]. 

While the k-prototypes method for clustering used in the analysis has the advantage of being able to manage mixed-data types (numerical and categorical variables), it is subject to the same limitations as other clustering methods in that it will statistically provide clusters where relationships between variables are identified regardless of whether they are theoretically meaningful. Therefore, the selection of theoretically appropriate variables and the interpretation of the clusters remains the responsibility of the researchers. Similarly, use of the elbow method for deciding how many clusters provide the most meaningful solution involves a degree of researcher interpretation in identifying the elbow, particularly if the scree plot curve does not show an obvious the point of inflexion (elbow). Again, this requires an understanding of the data and what is theoretically plausible to interpret as the optimum number of clusters and in this study the elbow appeared most prominent at six clusters.

## Conclusion

The findings highlight the relevance of specific individual characteristics in determining the risk of overweight and obesity, and differences in the extent of the relationship to poorer overall health, as well as other conditions specifically associated with obesity. This understanding should ensure that within an overall systems based approach to tackling this major public health issue, there is adequate recognition of the complexity of the explanatory factors driving inequalities. In turn this could lead to more specific tailored approaches to supporting weight management for different groups.

## Data Availability

Applications to access the data file and R code may be made to the data owners, the Sheffield Centre for Health and Related Research, University of Sheffield, 30 Regent Street, Sheffield, S1 4DA, UK (Professor Elizabeth Goyder, e.goyder@sheffield.ac.uk).
